# Dose-effect of exercise intervention on heart rate variability of acclimatized young male lowlanders at 3,680 m

**DOI:** 10.3389/fphys.2024.1331693

**Published:** 2024-03-27

**Authors:** Rui Su, Ping Peng, Wenrui Zhang, Jie Huang, Jing Fan, Delong Zhang, Jiayuan He, Hailin Ma, Hao Li

**Affiliations:** ^1^ Key Laboratory of High Altitudes Brain Science and Environmental Acclimation, Tibet University, Lhasa, China; ^2^ School of Psychological and Cognitive Sciences and Beijing Key Laboratory of Behavior and Mental Health, Peking University, Beijing, China; ^3^ Key Laboratory of Brain, Cognition and Education Sciences, Ministry of Education, Guangzhou, China; ^4^ School of Psychology, Center for Studies of Psychological Application, and Guangdong Key Laboratory of Mental Health and Cognitive Science, South China Normal University, Guangzhou, China; ^5^ National Clinical Research Center for Geriatric, West China Hospital, Sichuan University, Chengdu, Sichuan, China; ^6^ Med-X Center for Manufacturing, Sichuan University, Chengdu, Sichuan, China

**Keywords:** aerobic exercise, exercise intensity, high altitude, heart rate variability, lowlanders

## Abstract

This study investigated whether exercise could improve the reduced HRV in an environment of high altitude. A total of 97 young, healthy male lowlanders living at 3,680 m for >1 year were recruited. They were randomized into four groups, of which three performed—low-, moderate-, and high-intensity (LI, MI, HI) aerobic exercise for 4 weeks, respectively. The remaining was the control group (CG) receiving no intervention. For HI, compared to other groups, heart rate (*p* = 0.002) was significantly decreased, while standard deviation of RR intervals (*p* < 0.001), SD2 of Poincaré plot (*p* = 0.046) and the number of successive RR interval pairs that differ by > 50 ms divided by total number of RR (*p* = 0.032), were significantly increased after intervention. For MI, significantly increase of trigonometric interpolation in NN interval (*p* = 0.016) was observed after exercise. Further, a decrease in systolic blood pressure (SBP) after high-intensity exercise was found significantly associated with an increase in SD2 (*r* = – 0.428, *p* = 0.042). These results indicated that there was a dose effect of different intensities of aerobic exercise on the HRV of acclimatized lowlanders. Moderate and high-intensity aerobic exercise would change the status of the autonomic nervous system (ANS) and decrease the blood pressure of acclimatized lowlanders exposed to high altitude.

## 1 Introduction

When traveling to high altitude, the decrease in the barometric pressure leads to a lower ambient partial pressure of oxygen (PO_2_), which results in various physiological changes in the body (de Aquino Lemos et al., 2012; Allwood et al., 2018). The basal autonomic tone compensatory changes by the reduced partial pressure of atmospheric oxygen at high altitude, as indicated by an increased heart rate (HR) and hyperventilation (Bärtsch and Gibbs, 2007; Favret and Richalet, 2007). Hypoxia and low pressure at high altitude stimulate chemoreceptors and baroreceptors, resulting in the activation of the sympathetic nervous system and renin-angiotensin system (Hou et al., 2023). This activation affects the sympathetic and vagus nerves innervating the heart, disrupting the balance of cardiac autonomic nervous system (ANS) regulation (Shen et al., 2017; Ando, 2018), which could be reflected by heart rate variability (HRV) (Oliveira et al., 2017). HRV represents the oscillations between successive heartbeats and is considered a non-invasive marker of the autonomic nervous control of the cardiovascular system (Akselrod et al., 1981).

Prolonged exposure to high-altitude environments can lead to an intensified respiration, a compromised circulation and a profound sympathetic-parasympathetic imbalance in individuals, which is manifested by decreased HRV (Malhotra et al., 1976; Dhar et al., 2014; Yang et al., 2022). Studies have shown that reduced HRV variability is a marker of inadequate acclimatization and physiological dysfunction (Zupet et al., 2009; Heathers, 2014; Dhar et al., 2018). Meanwhile, studies have found that reduced HRV at high altitude exposure is associated with altitude illnesses such as chronic high-altitude pulmonary hypertension (Karinen et al., 2012; Qian et al., 2020). Specifically, HRV is a predictor of altitude sickness incidence (Karinen et al., 2012). The sympathetic activation was associated with increased blood pressure in individuals at high altitude (Han et al., 2019) This result has also been confirmed in other populations, such as hypertensive patients and healthy individuals (Liao et al., 1996; Singh et al., 1998; Mussalo et al., 2001; Lutfi and Sukkar, 2012). Therefore, methods to help lowlanders cope with reduced HRV and better adaptive must be explored.

Physical exercise is currently considered a promising strategy to increase HRV (i.e., increase vagal-related HRV parameters during rest) (Navarro-Lomas et al., 2022). Exercise is recommended as an important strategy to improve HRV (Tseng et al., 2020). Normoxic exercise significantly improves post-exercise parasympathetic tone, enhances autonomic regulation of heart rate, reduces sympathetic tone, and increases HRV (Michael et al., 2017; Dias et al., 2021; Villafaina et al., 2021). Moreover, hypoxic exercise might promote greater physiological and health-related acclimatization compared to normoxic exercise (Görgens et al., 2017; Li et al., 2020). However, the effect of exercise intensity on HRV at high altitude may be completely different from that at sea level, given that the reduce exercise capacity of individuals at high altitude has been proven (Davis et al., 2015).

HRV data on intensities of aerobic exercise at high altitude are scarce and the available data are incongruent. One study found that 12 weeks of moderate exercise at 3,000 m was more effective in improving HRV in older men than sea-level (Park et al., 2019). However, another study found moderate heart rate-matched hypoxic exercise did not appear to cause additional cardiac autonomic and physiological responses (Fornasiero et al., 2019). As such, the comparison of different exercise intensities in hypoxic environments would better guide exercise in lowlanders. Besides, most studies were performed in an artificial environment (hypobaric or normobaric chambers, tents, or face masks with reduced inspired oxygen), ignoring other features of the high altitude and thus suffering from poor ecological validity. Therefore, the HRV data from a real high altitude is important for investigating the effects of different exercise intensities on individuals.

The aim of this study was to investigate the effect of aerobic exercise intervention on HRV (including time domain, frequency domain, and non-linear indicators) in acclimatized lowlanders. Gender and age significantly influence the changes in exercise-induced dependent variables (HRV indicators) (Grant and Janse van Rensburg, 2013). To avoid confounding the effects of hypoxic exercise by gender and age factors, only young males were considered in this study. HRV was measured from young males acclimatized to high altitude of 3,680 m after low, moderate, and high-intensity exercise interventions. In the present study, we hypothesized that HRV increases in lowlanders after moderate-intensity exercise intervention. The results would bridge a significant gap in the literature by revealing the response of different intensities of exercise to HRV.

## 2 Methods

### 2.1 Participants

**FIGURE 1:**
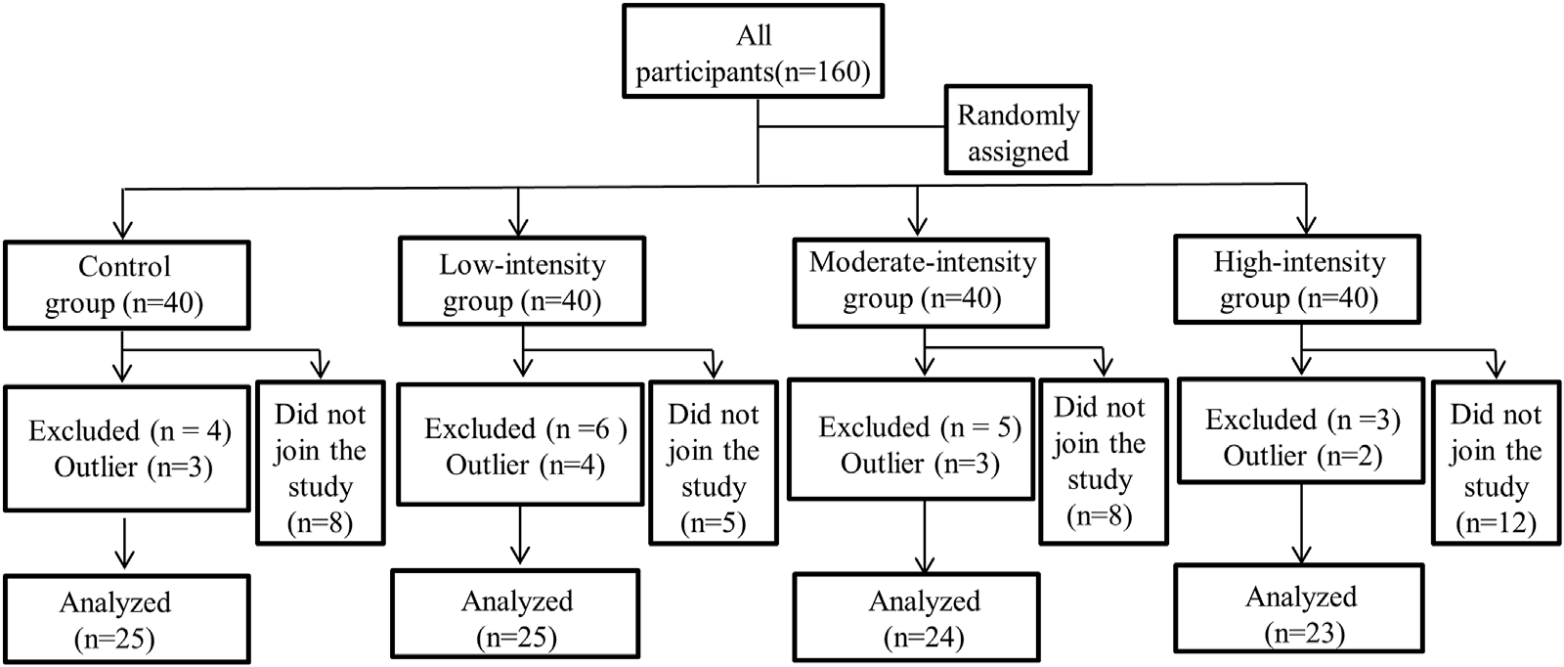
Study profile: Flow chart of the participants depicting recruitment, assessment, and retention in the study.

A total of 160 male university students who had lived in Lhasa (3,680 m, PO_2_ = 103mmHg, PB = 642.2 hPa) for >1 year (1.67 ± 0.41 years) were randomly divided into four groups: low-intensity exercise (LI) group; medium-intensity exercise (MI) group; high-intensity exercise (HI) group; and control group (CG; no exercise) (Figure 1). Participants were born at low altitude (<1,000 m) and had not visited highlands before entering college (defined as lowlanders). According to the findings of the Zubieta-Calleja study, the participants in this research have already acclimatized to high altitude (Zubieta-Calleja et al., 2007). On recruitment, questionnaires obtained information on participant disease history and contraindications. Participants with conditions that contraindicated exercise were excluded. Before the experiment, 33 participants dropped out of the experiment due to planning conflicts. During the experiment, 18 participants prematurely terminated exercise because of knee pain and panic attack. Data from 12 participants were excluded because of Electrocardiography (ECG) abnormalities or discontinuities. The number of participants in the final data analysis was 97 (mean age: 21.1 ± 1 year; height: 174.1 ± 5.9 cm; weight: 64.5 ± 9.2 kg). The research was conducted in compliance with ethical standards as verified by the ethics committee of Tibet University (XZTU2021ZRG-06) except for registration in a database. All study participants provided informed consent. All the procedures performed adhered to the tenets of the Declaration of Helsinki.

### 2.2 Study design

Participants were initially familiarized with the experimental procedures and equipment on 1 week, prior to the experiment. Participants were instructed to refrain from drinking tea, coffee, alcohol, or drugs that might excite the nervous system, and vigorous exercise 24 h before the experiment. All procedures were performed in Lhasa at a temperature of 22°C ± 1°C and humidity of 20% ± 5%.

During the baseline, participants underwent a basic physiological test, ECG, and cardiopulmonary exercise testing (CPET). Basic physiological tests included blood pressure (BP, mmHg) and oxygen saturation (SpO_2_). The CPET equipment was cycle ergometer (EC3000e, Ergoline GmbH, Bitz, Germany). The CPET procedure is consistent with previous study (Su et al., 2022). The post-intervention was conducted 24 h after the end of the exercise intervention period, and the test procedure was the same as that of the baseline.

Upon arrival at the laboratory, participants first rested for 2 minutes and then began the physiological measurements. SpO_2_ was measured with a pulse oximeter (YX303, Yuwell, Jiangsu, China) on the finger. BP was measured using an electronic sphygmomanometer (HEM-7136, Omron, Healthcare Inc.^®^, Kyoto, Japan).

After basic physiological measures, we measured the resting ECG of the participants to obtain HRV-related data. The participants were instructed to maintain a relaxed posture and breathe regularly and quietly during the experiment. Subsequently, the MP150 (BIOPAC^®^ Systems, Inc., Goleta, California, United States) analysis system, with a sampling frequency of 1,000 Hz, recorded ECG signals for 10 min. To avoid circadian influences, experiments were conducted at similar stages of the human physiological cycle: 8–9 a.m. and 4–5 p.m. For baseline, all participants were randomly assigned to these two periods. The test time and ECG for the post-intervention was the same as the baseline.

After baseline components, the participants were intervened with aerobic exercise for 20 min a day, 5 days a week, across 4 weeks (20 times in total, during autumn, September 2021). Exercise intensity levels in three groups were defined using HR reserve (HRR) according to formal guidelines. HRR was calculated by subtracting the resting HR (HR_rest_) from the maximum HR (HR_max_), where HR_max_ is measured by CPET in baseline. HR_rest_ is the average heart rate recorded during the ECG test. The participant’s HR_max_ was measured when they reach exhaustion and cannot continue anymore. The target HRs for three intensities of exercise were calculated based on the HRR: the target HR for low-intensity exercise was an HRR of 30%–39%, 40%–59% for moderate-intensity, and 60%–89% for high-intensity (Garber et al., 2011; Su et al., 2022). Daily exercise from 9 a.m. to 9 p.m. was conducted on a treadmill (GF9333, GFAMILY, Shenzhen, Guangdong, China). During the exercise, the participant’s HR was detected in real-time using a HR detector (Polar OH1, Polar ElectroOy, Kempele, Finland), which controls the HR indicated in the target HR range for the specific exercise intensity.

### 2.3 HRV data analysis

A standard collection of 5 min period was used to assess HRV (Malik, 1996). Then, a Butterworth filter was applied to the ECG signals, each QRS complex was identified using the P&T method (Pan–Tompkins algorithm), and the RR interval was calculated (Salsekar and Wadhwani, 2012). Finally, the time and frequency domains and non-linear indicators of HRV were calculated using MATLAB’s (Mathworks, Natick, MA, ver. 2016b) code-related programs (Vollmer, 2019).

HRV was analyzed with time domain, frequency domain, and nonlinearity indicators (Xhyheri et al., 2012; Shaffer and Ginsberg, 2017; Chen et al., 2020; He and Jiang, 2023). The specific physiological effects of the relevant indicators are shown in Table 1.

**TABLE 1:** Summary of the HRV indicators used in this study.

Indicators	Units	Description	Physiological significance
MeanRR	ms	The mean RR interval	The length of two adjacent sinus cardiac cycles
MeanHR	beats/min	The mean heart rate	The mean number of heartbeats per minute
SDNN	ms	Standard deviation of RR intervals	The SDNN represents total alteration
TINN	ms	Trigonometric interpolation in NN interval	The TINN indicates functional state of autonomic nerves
pNN50	%	The number of successive RR interval pairs that differ by > 50 ms divided by total number of RR	The pNN50 is indicative of vagal action
LF	ms^2^	Low frequency (0.04–0.15 Hz)	LF possibly correlated to sympathetic tone or to autonomic balance
HF	ms^2^	High frequency (0.15–0.40 Hz)	HF is considered to the activity of the parasympathetic system (vagus nerve)
LF/HF	-	Ratio LF [ms^2^]/HF [ms^2^]	LF/HF ratio is considered to show sympathovagal balance
TP	ms^2^	Total power spectral area (00.50 Hz)	TP is a broad measure of autonomic activity
SD1	ms	Standard deviation of the Poincaré plot perpendicular to the identity line	SD1 reflect the short-term dynamics of HRV
SD2	ms	Standard deviation of the Poincaré plot along to the identiy line	SD2 reflect the long-term dynamics of HRV
SampEn	-	Sample entropy	It quantifies the complexity in signals
ApEn	-	Approximate entropy	It measures the regularity and complexity of a time series

### 2.4 Statistical analysis

A linear mixed model (LMM) was used to test the significance of the effect of group (LI vs MI vs HI vs CG), time (Baseline vs After), and their interaction over the course of the study. In all models, the participants were considered random effects to account for within-subject correlations over time. Group was used as a fixed factor, and time was used as a repeated measure. The model specifilication was as follows: Conc ∼ Time + Group + Group * Time + (1| Participants). *Post hoc* comparisons were performed using Tukey test. The threshold for statistical significance was set at 5%, and the Satterthwaite approximation was used to compute the degrees of freedom in the denominator of the LMM. Correlation analysis was used to examine the relationship between HRV and basic physiological measurements. All statistical tests were conducted with R software (R Statistical Software, R Foundation for Statistical Computing, Vienna, Austria).

## 3 Results

### 3.1 Baseline characteristics

The baseline demographic, physiological and CPET data of the groups included in the study are shown in Table 2. There were no differences in basic demographic information between the four groups except for age, which was higher in the MI group than the HI and CG. In basic physiological data, the systolic blood pressure (SBP) was significantly higher in the MI as well as HI groups than in the CG group.

**TABLE 2:** Baseline characteristics of groups included in the study.

	LI	MI	HI	CG	*p*-values
Age (years)	21.29 (1.09)	21.72 (1.00)^a^ ^,^ ^d^	20.73 (1.09)	20.88 (1.02)	0.008
Bodyweight (kg)	64.52 (7.44)	68.625 (9.41)	61.96 (8.11)	63.22 (10.67)	0.067
Body height (m)	1.73 (0.046)	1.76 (0.059)	1.74 (0.047)	1.72 (0.074)	0.117
BMI (kg/m^2^)	21.41 (2.48)	22.02 (2.47)	20.38 (2.73)	21.27 (3.21)	0.242
Body temperature (°C)	36.43 (0.40)	36.29 (0.48)	36.52 (0.34)	36.34 (0.44)	0.258
**CPET index**					
HR_max_ (bpm)	174.44 (15.94)	175.67 (12.36)	179.57 (9.26)	177.40 (10.95)	0.405
${\dot{V}O}_{2\max}$ (mL/min)	35.25 (6.78)	34.88 (5.92)	38.04 (7.74)	36.48 (5.08)	0.252
${\dot{V}E}_{\max}$ (L/min)	106.79 (30.59)	116.42 (28.68)	114.39 (17.95)	115.6 (22.23)	0.531
BF_max_ (cpm)	53.36 (11.07)	51.92 (11.19)	53.26 (10.81)	48.25 (8.09)	0.288

Data are presented as mean (standard deviation).

BMI: body mass index; HR_max_: maximum heart rate; ${\dot{V}O}_{2\max}$ : maximal oxygen uptake; ${\dot{V}E}_{\max}$ : maximum ventilation; BF_max_: maximum breathing frequency.

*P*-value refers to results of comparison of different groups. Differences for effects of exercise intensities:

^a^ Difference from CG group

^b^ Difference from LI group

^c^ Difference from MI group

^d^ Difference from HI group.

### 3.2 Basic physiological

In the basic physiological analysis, we found a main effect for “Time” for both SBP and DBP (*p* < 0.001; *p* = 0.012). At the same time, a main effect was observed for “Group” in SBP (*p* = 0.025). In the HI and MI groups, SBP was significantly lower (*p* = 0.007 and *p* = 0.002) when compared with the baseline value. This is described in more detail in Table 3.

**TABLE 3:** Physiological measures of different exercise intensity groups.

		Groups				*p*-values		
Outcome	Time period	LI	MI	HI	CG	“Group * time”	“Time”	“Group”
**Basic physiological index**								
SBP	Baseline	118.56 (17.98)	123.18 (11.90)	123.60 (13.4)	112.62 (11.93)	0.542	<.001	0.025
	After	112.97 (11.96)	114.56 (10.55)	113.56 (9.60)	108.01 (14.03)			
DBP	Baseline	77.68 (12.27)	82.54 (11.64)	79.30 (7.84)	77.00 (6.37)	0.253	0.012	0.433
	After	74.74 (8.54)	74.42 (10.51)	77.72 (7.58)	75.58 (9.19)			
SpO_2_	Baseline	89.28 (4.37)	90.08 (4.86)	90.91 (2.50)	91.62 (2.51)	0.080	0.113	0.256
	After	88.78 (3.41)	91.25 (3.13)	89.45 (4.75)	88.75 (5.20)			

Basic physiological index at baseline as well as at post-intervention time in the low-intensity (LI), moderate-intensity (MI), and high-intensity (HI) exercise groups.

SBP: systolic blood pressure; DBP: diastolic blood pressure; SpO_2_, pulse oximeter oxygen saturation.

Data are presented as mean (standard deviation). *p*-values from the main effects linear mixed models.

### 3.3 HRV indicators

Table 4 summarizes the HRV outcome data at baseline and after exercise for each group.

**TABLE 4:** Comparison of HRV in different exercise intensity groups.

		Groups				*p-values*		
Outcome	Time period	LI	MI	HI	CG	“Group * time”	“Time”	“Group”
**Time-domain analysis**								
MeanRR	Baseline	0.73 (0.1)	0.74 (0.12)	0.68 (0.06)	0.73 (0.08)	0.003	0.029	0.679
	After	0.73 (0.09)	0.76 (0.09)	0.77 (0.11)	0.71 (0.09)			
MeanHR	Baseline	83.55 (12.4)	83.41 (14.93)	88.39 (8.43)	82.63 (9.31)	0.005	0.027	0.814
	After	83.16 (10.11)	79.65 (10.81)	78.77 (11.80)	85.41 (11.6)			
SDNN	Baseline	59.0 (19.06)	53.07 (21.06)	53.42 (24.24)	59.21 (21.84)	0.047	0.006	0.708
	After	66.09 (33.94)	67.54 (24.59)	70.56 (24.52)	54.27 (20.4)			
TINN	Baseline	19.2 (7.3)	15.7 (6.1)	16.2 (5.4)	18.4 (6.1)	0.012	0.025	0.879
	After	18.8 ((6.9)	20.4 (8.1)	19.6 (7.8)	17.3 (5.6)			
pNN50	Baseline	12.4 (12.7)	10.3 (9.7)	7.2 (5.9)	14.3 (13.9)	0.029	0.112	0.94
	After	14 (15.5)	14.7 (15.2)	15.5 (16.5)	9.8 (9.2)			
**Frequency-domain analysis**								
LF	Baseline	1,337 (504)	1,343 (443)	1,207 (393)	1,295 (317)	0.435	0.84	0.796
	After	1,342 (511)	1,191 (278)	1,301 (293)	1,306 (487)			
HF	Baseline	636 (435)	764 (368)	792 (394)	574 (291)	0.773	0.037	0.029
	After	841 (466)	854 (415)	900 (444)	627 (370)			
LF/HF	Baseline	3.47 (2.69)	2.35 (1.87)	2.29 (2.88)	3.1 (2.08)	0.556	0.985	0.121
	After	2.89 (3.22)	2.10 (1.9)	1.98 (1.44)	4.35 (8.5)			
TP	Baseline	1973.74 (541.18)	2,106.89 (441.72)	1999.43 (602.38)	1869.60 (360.4)	0.461	0.879	0.899
	After	2,182.11 (431.98)	2045.48 (372.88)	2,201.79 (459.65)	1932,38 (560.05)			
**Nonlinear analysis**								
SD1	Baseline	32.72 (16.9)	33.42 (19.67)	34.8 (27.44)	33.86 (20.93)	0.239	0.008	0.388
	After	45.43 (38.57)	43.7 (19.3)	48.54 (27.24)	31.21 (18.96)			
SD2	Baseline	75.7 (25.22)	66.77 (24.49)	65.9 (24.57)	75.86 (25.72)	0.013	0.007	0.858
	After	80.08 (33.47)	84 (30.43)	86.08 (26.48)	69.18 (24.35)			
SampEn	Baseline	1.24 (0.27)	1.24 (0.38)	1.25 (0.4)	1.35 (0.37)	0.874	0.577	0.808
	After	1.25 (0.38)	1.25 (0.34)	1.21 (0.35)	1.26 (0.33)			
ApEn	Baseline	1.07 (0.1)	1.04 (0.19)	1.07 (0.23)	1.1 (0.18)	0.948	0.612	0.633
	After	1.05 (0.17)	1.06 (0.16)	1.04 (0.16)	1.08 (0.16)			

HRV, at baseline as well as at post-intervention time in the low-intensity (LI), moderate-intensity (MI), and high-intensity (HI) exercise groups.

Data are presented as mean (standard deviation). *p-*values from the main effects linear mixed models.

#### 3.3.1 Time domain analysis

In the time domain analysis, we found a significant “Group * Time” interaction (*p* = 0.003; *p* = 0.005) and a main effect for “Time” (*p* = 0.029; *p* = 0.027) for both mean RR interval (MeanRR) and mean heart rate (MeanHR). This is described in more detail in Table 4. In the HI group, MeanHR was significantly lower (*p* = 0.002) and MeanRR was significantly higher (*p* < 0.001) when compared with the baseline value. MeanHR was significantly lower and MeanRR was significantly higher in the HI group than in the CG group (*p* = 0.044; *p* = 0.026) following exercise. This suggests that high intensity exercise significantly increased the RR interval and reduced individual heart rate.

To further probe the effect of different exercise intensities, we next analyzed the standard deviation of RR intervals (SDNN), the baseline width of the RR interval histogram (TINN) and the number of successive RR interval pairs that differ by > 50 ms divided by total number of RR (pNN50). In Table 4, the interaction effect was observed for “Group * Time” (*p* = 0.047; *p* = 0.012; *p* = 0.029) for results including the domains (SDNN, TINN, pNN50). In the HI group, the pNN50 significant increases relative to baseline (*p* = 0.032) were observed. Significant increases in TINN in the MI groups (*p* = 0.016) were found, while the other groups were not significantly different from baseline after exercise. There was no difference between groups after exercise except for SDNN. We found that SDNN was significantly higher in the HI group than CG (*p* = 0.02).

#### 3.3.2 Frequency domain analysis

In Table 4, main effect was observed for “Time” (*p* = 0.037) and “Group” (*p* = 0.029) for results including the high frequency (HF). We found that HF was significantly higher in both HI and MI than in the CG group following exercise (*p* = 0.019; *p* = 0.048). There were no main effect observed either for “Time,” “Group,” or for the “Group * Time” interaction for results including the domains (Low frequency, Low frequency/High frequency, Totalpower).

#### 3.3.3 Nonlinear analysis

**FIGURE 2:**
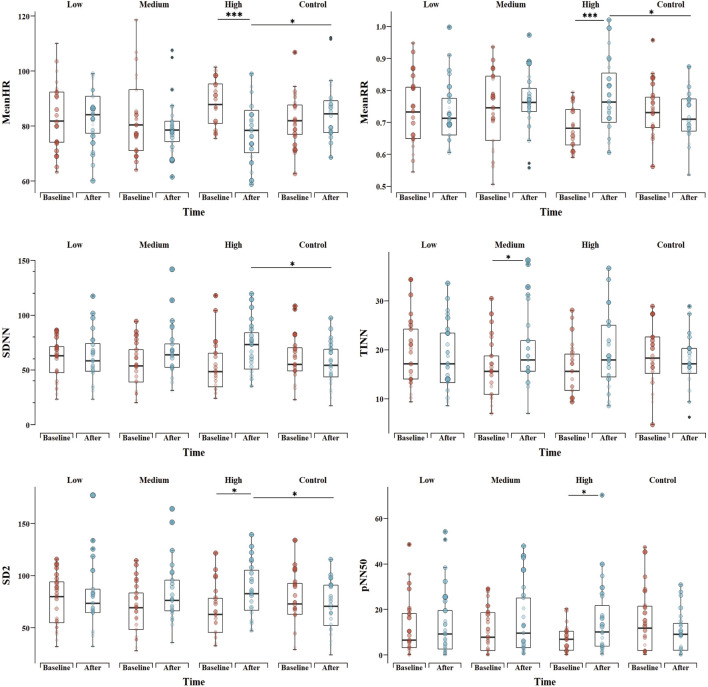
Comparison of HRV indicators, including mean heart rate (MeanHR), mean RR interval (MeanRR), standard deviation of RR intervals (SDNN), the baseline width of the RR interval histogram (TINN), the number of successive RR interval pairs that differ by > 50 ms divided by total number of RR (pNN50) and SD2 between groups. *p < 0.05, **p < 0.01, ***p < 0.001 for difference with Baseline vs. After and difference between groups in After.

The SD2 results show significant “Group * Time” interaction (*p* = 0.013) and “Time” (*p* = 0.007) effects (Table 4). In the HI group, SD2 increased significantly (*p* = 0.046) and was significantly higher than CG group after the exercise (*p* = 0.032). The results show a main effect for “Time” (*p* = 0.008), and there was no difference between the “Groups” or for the “Group * Time” interaction for the SD1. As for sample entropy (SampEn) and approximate entropy (ApEn), no main effect was observed either for “Time,” “Group,” or for the “Group * Time” interaction. The comparison of HRV indicators, including MeanHR, MeanRR, SDNN, TINN, pNN50 and SD2 among groups is shown in Figure 2.

### 3.4 Correlation analyses

The possible relationship between basic physiological changes and HRV indicators between different groups was explored. Correlation analysis showed a significant negative correlation between SBP and changes (After - Baseline) in SDNN, TINN, pNN50, SD1, SD2, and HF in the HI group as shown in the table, while no significant correlation was found in the other groups (Table 5).

**TABLE 5:** Correlation analysis between changes (After - Baseline) in basic physiological data and HRV of HI group.

	1	2	3	4	5	6	7	8	9	10	11
1.SpO_2_	1										
2.SBP	−.2	1									
3.DBP	.15	.38	1								
4.MeanHR	.06	.25	−.31	1							
5.MeanRR	−.01	−.25	.33	−.98^***^	1						
6.SDNN	.11	−.5^*^	−.3	−.13	.13	1					
7.TINN	.02	−.47^*^	.09	−.55^*^	.56^**^	.59^**^	1				
8.pNN50	.05	−.42^*^	.24	−.76^***^	.79^***^	.5^*^	.79^***^	1			
9.HF	.19	−.46^*^	−.31	−.13	.12	.6^**^	.18	.4	1		
10.SD1	.01	−.44^*^	−.29	−.13	.11	.93^***^	.44^*^	.5^*^	.79^***^	1	
11.SD2	.12	−.42^*^	−.14	−.28	.28	.94^***^	.65^**^	.6^**^	.55^**^	.91^***^	1

^*^ Stands for *p* < 0.05, ** stands for *p* < 0.01, *** stands for *p* < 0.001.

## 4 Discussion

To the best of our knowledge, this is the first study to explore the effects of exercise intensity on the HRV of acclimatized male lowlanders in a real high-altitude environment. The dose effect of high-altitude exercise on HRV was revealed. The specific findings could be summarized as follows: 1) Low-intensity exercise did not improve HRV in lowlanders; 2) Moderate-intensity and high-intensity exercise were effective in improving the degree of HRV in terms of TINN and SD2 in lowlanders; 3) High-intensity exercise significantly increased HRV, which was associated with a decrease in SBP. The present study demonstrates that moderate-to high-intensity exercise improves HRV in individuals, which is significant in reducing the incidence of cardiovascular disease at high altitude as well as in promoting individual acclimatization to high altitude.

As altitude increases, arterial oxygen saturation decreases further and heart rate increases to compensate for the decrease in arterial oxygen, leading to a further dysregulation of sympathetic control of the individual’s heart rate, which may be alleviated by exercise (Aebi et al., 2020). High altitude has been shown to increase circulating levels of pro-inflammatory cytokines such as c-reactive protein and IL-6 (Hartmann et al., 2000). And exercise can exhibit anti-inflammatory effects by inducing anti-inflammatory cytokines and down-regulating toll-like receptor (TLR) signaling pathways, whereas a reduction in the inflammatory response can increase HRV (Li et al., 2020). Meanwhile, increased ANS function was highly correlated with endurance exercise performance. Hypoxia exercise under showed acclimatization such as enhanced oxygen delivery and utilization capacity, cardiopulmonary function, mitochondrial capacity, oxidative enzyme capacity, angiogenesis, and muscle buffering capacity (Park et al., 2022).

The findings of the present study suggest that there was a dose effect of exercise intensity on the improvement of HRV. The magnitude of individual HRV changes may be linked to the exercise-intervention intensity (Laoutaris et al., 2008). Here, moderate, and high-intensity aerobic exercise had a significant enhancement on HRV, while there was no significant improvement in the LI group. Farah et al. found a theoretical ‘threshold effect’ for the intensity of aerobic exercise training in eliciting improvements in HRV and cardiovascular fitness in a 6-month exercise intervention study (Farah et al., 2014). Additionally, low-intensity exercise may not reach the threshold to modify the ANS. During exercise, factors such as the exercise intensity and physiological environment interact to produce the overall homeostatic stress or “training load” of the session (Mann et al., 2014). In contrast, the relative disturbance of resting physiological and metabolic processes by stressors created by low-intensity exercise and high-altitude environments may not have been significant enough to cause changes in individual homeostasis. Thus, low-intensity exercise has no improving effects on HRV in lowlanders.

There was no change in RR interval in lowland individuals after moderate-intensity exercise, but the degree of HRV in terms of TINN was found to increase in this study. The TINN index is an indicator of overall ANS activity (de Rezende Barbosa et al., 2016). Moderate-intensity exercise has some beneficial effect on HRV in young healthy lowlanders in the highlands. A relatively short period of exercise (2 months) at moderate intensity is reportedly sufficient to induce significant changes in HRV in older adults (Ferreira et al., 2017). Moderate exercise under hypoxic can improve ANS function by promoting microcirculation and facilitating the function of oxygen and carbon dioxide exchange in tissues (Millet et al., 2016; Park et al., 2022). In addition, there is evidence that exercise training leads to normalization of other components of neurohumoral excitation (Gademan et al., 2007). Jurca et al. showed that moderate-intensity exercise can maintain ANS balance by remodeling central glutamatergic and gamma-aminobutyric acid nerves (Jurca et al., 2004). However, James et al. found no effect of moderate-intensity exercise on the sympathetic and parasympathetic nerves of the heart (James et al., 2012). This may be a difference caused by the high-altitude environment. Under hypoxic conditions, the pronounced autonomic response may be attributable to the hypobaric pressure (Aebi et al., 2020). At high altitude, the combination of hypobaric hypoxia with moderate exercise intensity creates a distinct intensity exercise stressor, creating a tendency for individuals to adapt to high-intensity stressors.

In the present study, only high-intensity exercise reduced MeanHR and increased MeanRR in lowlanders. We observed a significant increase in the time domain and non-linear indicators after high-intensity exercise. The increase in SD2 and pNN50 reflects the increased activation of the parasympathetic nerve activity (PNA) (Xhyheri et al., 2012). After intervention, HF in the moderate-intensity and high-intensity groups was significantly higher than that in the control group. HF also indicates the activity of the parasympathetic system. We have demonstrated increased parasympathetic regulation of the heart after high-intensity exercise in correlation analysis results. Kim et al. found an increase in ANS function in amateur male swimmers after 6 weeks of hypoxic high-intensity exercise (Kim et al., 2021). High-intensity exercise may effectively alter cardiac function by activating beta-adrenergic receptors in the myocardium, improving the viability of the PNS and increasing venous return in a hypoxia environment (Park et al., 2018). Moreover, high-intensity or prolonged exercise under demanding conditions may lead to an acute decline in cardiac function, which may affect HRV (Kleinnibbelink et al., 2021). However, in this study, aerobic exercise was performed at a relative intensity, i.e., as a percentage of an individual’s reserve heart rate, and not at an absolute intensity (Fornasiero et al., 2019). At the same absolute exercise intensity as the plains, such as walking exercise at 100W, hypoxia increases individual physiological and perceptual responses (Li et al., 2022). Therefore, the high intensities in this study were not stronger compared to the lower altitude.

One effect of increased sympathetic adrenal activity in response to hypoxia exposure is an increase in mean arterial pressure, which may lead to systemic hypertension (Wu et al., 2007). Therefore, BP monitoring is necessary for young lowlanders exposed to high altitude (Siqués et al., 2009). This study found a positive correlation between the increase in HRV and the decrease in SBP after high-intensity exercise. Indeed, nearly half of the participants in the high-intensity training group had systolic blood pressure reductions of >10 mmHg, which can have significant clinical benefits. Beneficial effects of regular hypoxic training on blood pressure regulation have been observed in some studies. Kong et al. and Morishima et al. reported a 10 mmHg and 7 mmHg decrease in systolic blood pressure after 4 weeks exercise training at 16.4%–14.5% FiO_2_ (Kong et al., 2014; Morishima et al., 2015)*.* It is possible that the vagus nerve induces vasodilation by increasing vasoactive intestinal peptides, thereby elevating coronary blood flow (Feliciano and Henning, 1998). This reveals that a rise in HRV after exercise may be an important target for ameliorating elevated BP in a high-altitude environment. However, this requires further confirmation.

There were several limitations in this study. First, the data were all from young males, ignoring the effect of gender on HRV at high altitude. Females have been found to have relatively higher SpO_2_ and estrogen than males, which was reported to provide greater resistance to hypoxia. To avoid confounding the effects of hypoxic exercise by gender factors, only males were considered in this study (Heyer et al., 2005; Jung et al., 2020). However, female data was important and would be definitely investigated in future studies. Secondly, there were differences in age in the baseline data. Considering the mean age difference between groups was <1 year, the effect was little. Thirdly, the breathing patterns of the participants were not well controlled. However, considering that these participants had regular breathing and no deep breathing, the effect of breathing on HRV was minimal. We will incorporate electrocardiogram-derived respiration (EDR) to better explore the relationship between HRV and respiration (Sakai et al., 2019). Finally, the intervention period of this study was only 4 weeks, with each exercise session lasting only 20 min, both of which could impact the effectiveness of aerobic training on HRV. Future studies can use varying intervention durations and exercise durations to further explore the optimal conditions for enhancing HRV through aerobic exercise. Meanwhile, we will expand the study population to further explore the physiological mechanisms of HRV following exercise and the optimal combination between physical activity (e.g., intensity of exercise, type of activity) and hypoxic (e.g., altitude level) components.

## 5 Conclusion

The findings of this study point to a dose effect on HRV in acclimatized lowlanders after 4-week aerobic exercise of different intensities. HRV increased and BP decreased at moderate intensity compared to low intensity. The 4 weeks of high intensity exercise significantly changes the activation status of parasympathetic nerves, which in turn increases HRV, but is accompanied by a decrease in BP, which requires further investigation into the underlying mechanisms. Caution is needed at very high altitude for high-intensity exercise and to assess the applicability of our findings to other populations or exercises.

## Data Availability

The raw data supporting the conclusions of this article will be made available by the authors, without undue reservation.
